# The Role of Eicosanoids in Alzheimer’s Disease

**DOI:** 10.3390/ijerph16142560

**Published:** 2019-07-18

**Authors:** Roger G. Biringer

**Affiliations:** College of Osteopathic Medicine, Lake Erie College of Osteopathic Medicine, 5000 Lakewood Ranch Blvd., Bradenton, FL 34211, USA; rbiringer@lecom.edu; Tel.: +1-941-782-5925

**Keywords:** Alzheimer’s disease, eicosanoid, prostaglandin, thromboxane, leukotriene, lipoxin, resolving, protectin, maresin, isoprostane

## Abstract

Alzheimer’s disease (AD) is one of the most common neurodegenerative disorders known. Estimates from the Alzheimer’s Association suggest that there are currently 5.8 million Americans living with the disease and that this will rise to 14 million by 2050. Research over the decades has revealed that AD pathology is complex and involves a number of cellular processes. In addition to the well-studied amyloid-β and tau pathology, oxidative damage to lipids and inflammation are also intimately involved. One aspect all these processes share is eicosanoid signaling. Eicosanoids are derived from polyunsaturated fatty acids by enzymatic or non-enzymatic means and serve as short-lived autocrine or paracrine agents. Some of these eicosanoids serve to exacerbate AD pathology while others serve to remediate AD pathology. A thorough understanding of eicosanoid signaling is paramount for understanding the underlying mechanisms and developing potential treatments for AD. In this review, eicosanoid metabolism is examined in terms of in vivo production, sites of production, receptor signaling, non-AD biological functions, and known participation in AD pathology.

## 1. Introduction

Alzheimer’s disease (AD) is the primary neurodegenerative disorder causing dementia in the elderly. The primary pathogenic signature is the extracellular deposition of beta amyloid protein (Aβ) and the intracellular accumulation of tau protein. Aβ is a 36–42 residue proteolytic product formed by β- and γ-secretase cleavage of the amyloid precursor protein (APP) that ultimately precipitates as amyloid fibrils. Prior to aggregation, APP is known to complex with Zn^2+^, Fe^3+^, and Cu^2+^ which then mediates free radical formation and subsequent neural damage. The tau protein’s main function in neurons is to modulate the stability of microtubules in axons. However, upon hyper-phosphorylation of tau, self-assembly occurs and insoluble aggregates form that block synapses and nutrient uptake, ultimately leading to neuronal death.

Eicosanoids are amphipathic, bioactive signaling molecules derived from the oxidation of arachidonic acid (AA) and other similar polyunsaturated fatty acids (PUFAs). There are multiple subfamilies of eicosanoids, including the prostaglandins, thromboxanes, leukotrienes, lipoxins, resolvins, protectins, maresins, isoprostanes, endocannabinoids, HETEs, and eoxins to name a few. Although the biological functions of eicosanoids cover a wide range, one thing many have in common is context dependency. That is, stimulation by a particular eicosanoid in one cell or tissue may in fact elicit a different and, at times, opposite effect in another cell or tissue. Eicosanoids are key paracrine and autocrine biochemical signaling molecules in the central nervous system (CNS) and are reported to be involved in numerous processes including memory and learning [1], cerebral blood flow [2], sleep [3], as well as regulation of neuroinflammation [1]. The fact that each of these neurological functions has been shown to be involved in AD etiology [4,5,6,7] strongly suggests the involvement of eicosanoid signaling in the disease.

## 2. Neuroinflammation

Up until the mid-1990s the central nervous system (CNS) was deemed to be “immune privileged”, that is, devoid of inflammation. However, since that time, multiple studies have shown that neuroinflammation is involved in numerous CNS disorders, including AD. See the review by Lucas et al. [8]. In particular, microglial cells, the resident macrophages of the brain, are known to produce the inflammatory cytokines IL-1, IL-6, and TNFα [9]. These cells are differentially expressed across brain regions and have been shown to correlate well with the distributions of AD related neuritic plaques [10,11]. Both APP and Aβ peptides are known to be strong glial activators and hence promote the release of these pro-inflammatory factors. These factors in turn are known to upregulate APP production as well as hyperphosphorylation of tau, thus amplifying the process [12]. Activated microglia are also reported to be major producers of the prostaglandin PGE_2_ through the COX-2 pathway [13] and serve as a trigger for uncontrolled astrocyte proliferation [14], another feature of AD [15]. See the recent review by Wang and Colonna [16]. 

## 3. Free AA and cPLA2-α in AD

Free AA ((5Z,8Z,11Z,14Z)-5,8,11,14-eicosatetraenoic acid) is not only a precursor to many classes of eicosanoids, some of which are directly involved in AD, but also is an active participant in synaptic functions. AA produced by the post-synaptic neuron has been shown to act as a retrograde messenger on the pre-synaptic membrane where it increases neurotransmitter release, resulting in the induction of long-term potentiation (LTP) [17,18]. Further, AA has also been shown to facilitate inhibition of voltage-gated K^+^ channels on the presynaptic membrane [19] and activate SNARE receptors [20], both of which are known to enhance neurotransmitter release. In contrast, treatment of mouse brains with human Aβ oligomers has been shown to inhibit LTP [21]. At face value, the actions of AA and Aβ appear juxtaposed. However, it has been proposed that excessive neurotransmitter release and excessive maintenance of LTP have detrimental effects on memory storage, thus exacerbating memory issues in AD patients [22]. Additionally, PET scans of AD patients and controls administered radiolabeled AA revealed that AA accumulation in AD brains was elevated compared to controls (26% increase in brain radioactivity normalized to integrated plasma radioactivity) and was particularly elevated in regions with a high density of neuritic plaques with activated microglia [23].

The phospholipase A_2_ (PLA_2_) family catalyzes the cleavage of fatty acids from glycerophospholipids at the *sn-2* position, where AA, if present, is almost exclusively esterified. Hence, this class of enzyme provides AA for the production of eicosanoids involved in AD. The 22 known PLA_2_s are further classified into one of three categories [24]: cytosolic Ca^2+^-dependent PLA_2_ (cPLA_2_), secretory Ca^2+^-dependent PLA_2_ (sPLA_2_), and cytosolic Ca^2+^-independent PLA_2_ (iPLA_2_). Both sPLA_2_ [25] and cPLA_2_ [22] were found to be upregulated in AD whereas iPLA_2_ was downregulated [26]. Since the latter is associated with housekeeping functions with a low preference for AA, its downregulation in AD makes sense. sPLA_2_, in particular the isoform sPLA_2_-IIA, was found to be up-regulated in astrocytes cultured from AD post-mortem human brains compared to elderly non-demented brains and was found associated with Aβ-containing plaques [25]. This up-regulation was facilitated by both Aβ_1–42_ and IL-1β, each of which is known to be associated with plaque formation in AD. In a transgenic murine model, expression of AD-mutant human amyloid precursor protein (hAPP) resulted in an increased production of AA in mouse neuronal cultured brain [27]. The increased production of AA was apparently caused by phosphorylation and subsequent activation of cytosolic GroupIV-PLA2 in hAPP mice as compared to controls; GroupIV-PLA2 has a marked preference for AA. Treatment of murine brain cell cultures with synthetic Aβ_1–42_ also facilitated phosphorylation/activation of GroupIV-PLA2 and treatment with either Aβ_1–42_ or AA caused a dose-dependent reduction in neuronal viability. Further, treatment of rat cortical neuron cultures with a non-specific PLA_2_ inhibitor has been shown to reduce the production of AA and Aβ_1–40_- as well as Aβ_1–42_-induced neuron apoptosis [28]. Clearly, there is a yet-to-be understood synergy between AA, its production by sPLA_2_, and amyloid peptides in AD progression.

## 4. Cyclooxygenases in AD

**Figure 1:**
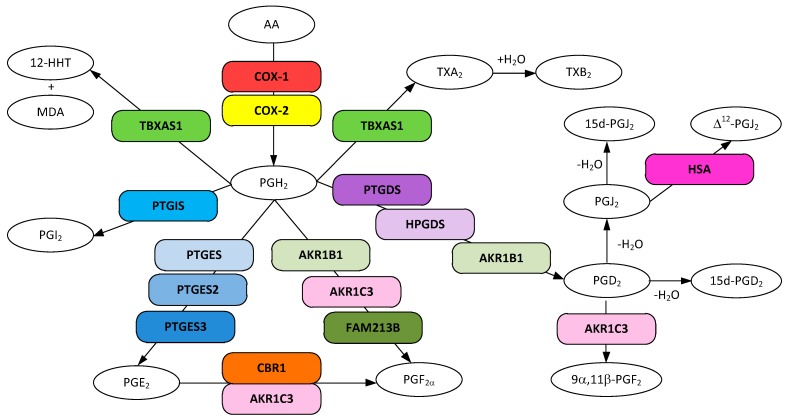
Metabolic pathways for prostanoid and thromboxane biosynthesis from arachidonic acid. Gene designations are given for all but the COX participating enzymes (rounded boxes) and accepted acronyms given for metabolites (ovals).

Cyclooxygenases catalyze the first step in prostanoid biosynthesis whereby arachidonic acid is converted to the endoperoxide prostaglandin H_2_ (PGH_2_, (Z)-7-[(1R,4S,5R,6R)-6-[(E,3S)-3-hydroxyoct-1-enyl]-2,3-dioxabicyclo[2.2.1]heptan-5-yl]hept-5-enoic acid) via the transient prostaglandin G_2_ (PGG_2_, (Z)-7-[(1R,4S,5R,6R)-6-[(E,3S)-3-hydroperoxyoct-1-enyl]-2,3-dioxabicyclo[2.2.1]heptan-5-yl]hept-5-enoic acid) (Figure 1). PGH_2_ is a multifunctional metabolite and serves as a precursor for the enzymatic synthesis of other prostanoids: PGI_2_, PGE_2_, PGF_2_α, PGD_2_, and TXA_2_. PGH_2_ is a labile endoperoxide and it rapidly and non-enzymatically rearranges to both PGD_2_ and PGE_2_. There are two known protein isoforms of cyclooxygenase, prostaglandin G/H synthase 1 (COX-1) and prostaglandin G/H synthase 2 (COX-2). A third isoform (COX-3, COX-1b), a splice variant of COX-1, has been identified in humans and other mammals, but its involvement in human prostaglandin synthesis has largely been discounted [29]. In most tissues the COX-1 is expressed constitutively and functions to maintain the GI tract and renal function [30] whereas COX-2, normally present at undetectable levels in most cells, is induced during inflammation. In contrast, in the brain, testes, and the macula densa of the kidney, both isoforms were found to be expressed constitutively [31,32]. Both isomers have been found associated with endoplasmic reticulum (ER) and nuclear membranes [33]. Although COX-2 is expressed throughout the forebrain, the highest levels have been found in the hippocampus and cerebral cortex and located in particular groups of neurons, and are not typically found in glial cells [32] unless certain conditions prevail (e.g., chronic cerebral ischemia) [34]. 

Non-steroidal anti-inflammatory agents (NSAIDs) are known inhibitors of cyclooxygenases and there are numerous reports that show unequivocally that long term use of NSAIDs is associated with a reduction in the risk of developing AD [35,36]. For example, in a population-based cohort study of 6989 subjects 55 years or older living in Ommoord, Netherlands, long term use of NSAIDs (24 months or more) reduced the relative risk of AD by 80% [37]. Further, a six-month, double-blind, placebo-controlled study revealed that the non-selective COX inhibitor indomethacin (seven times more effective for COX-1 than COX-2) appeared to protect mild to moderately impaired Alzheimer’s disease patients from the degree of cognitive decline as compared to a placebo-treated group [38]. In fact, the treated group showed improved cognition. Not all studies have produced such positive results [39] and the effect may depend on the NSAID used and its target [35] as well as the stage of AD when the treatment was begun [40].

Much of the focus on the involvement of cyclooxygenases in AD has been directed to the COX-2 isoform. Semiquantitative immunostaining of AD brain and age matched controls have revealed elevated COX-2 in CA1–CA4 subdivisions of the hippocampal pyramidal layer in AD brain compared to controls and the observed COX-2 levels correlated with amyloid plaque density [41]. However, immunostaining of post-mortem AD and control brains revealed that COX-2 positive neurons decrease over the progression of the disease once the individual has reached a clinical dementia rating (CDR) of 5 [42]. Further, COX-2 positive neurons have been shown to decrease with increasing amyloid deposit accumulation except in the very early stages (Braak stage A) where there was a moderate, but significant increase [43]. In support of this observation, treatment of neuroblastoma cells with synthetic Aβ peptides in vitro has been shown to induce the expression of COX-2 [44]. These results show that COX-2 is involved in the early stages of AD, but is unlikely to be involved in the pathology in the end stage AD. In a mouse AD model, selective inhibition of COX-2 or combined inhibition of COX-1 and COX-2 prevented the suppression of LTP by Aβ and improved spacial memory, whereas selective inhibition of COX-1 showed no effect on LTP suppression [45]. The authors further suggested that it is the inhibition of COX-2 concentrated in dendritic spines that prevent the deleterious effects of Aβ in this region. These data also support the involvement of COX-2 in the early stages of AD with diminished effects in the latter stages.

Early work largely ignores the involvement of COX-1 in AD. This possibly due to the fact that COX-2 is a more pharmacologically advantageous target than COX-1, as COX-1 inhibition can cause severe side effects, including gastric damage. Although COX-1 may not be an opportune therapeutic target, its involvement in AD is clear. Immunostaining of human brain tissue obtained from both AD patients and controls revealed that microglia express COX-1 in AD and control brains, regardless of activation state [46]. However, in AD brains, activated microglia were found surrounding and imbedded in Aβ plaques, suggesting the production of eicosanoids in AD positive regions. Further support for the involvement of COX-1 comes from studies utilizing Triflusal, a specific, irreversible inhibitor of COX-1 [47] which has only a moderate effect on COX-2 expression (30% reduction) [48]. In a mouse AD model, Trifusal did not alter the total accumulation of Aβ, but significantly reduced (50%) the neuritic plaque load and associated glial cell proliferation [49]. Further, reductions in pro-inflammatory cytokine levels as well as improved cognition were also observed. These results and the aforementioned clinical trial with indomethacin [46], a preferential COX-1 inhibitor, support the involvement of COX-1 in both early and later stages of AD. The fact that selective inhibition of COX-1 had no effect on Aβ suppression of LTP and selective inhibition of COX-2 did [45] indicates a mechanism of action for COX-1 that differs from that for COX-2.

## 5. Prostaglandins, Thromboxanes, and Their Receptors in AD.

One of the earliest reports showing the involvement of prostaglandins and thromboxanes in AD was obtained from the analysis of the cortex of post-mortem brains from five patients with pathologically verified AD as compared to eight age-matched controls [50]. The results revealed that the amounts of prostaglandin PGD_2_ and the thromboxane TXB_2_ were significantly elevated in AD brains as compared to control brains, not surprising given that both are strong vasoconstrictors [5]. The fact that NSAIDS inhibit cyclooxygenases (COX-1 and COX-2), the enzymes that convert arachidonic acid (AA) to prostaglandin H_2_ (PGH_2_), the precursor to all prostanoids and thromboxanes (Figure 1), strongly indicates that one or more of these eicosanoids is involved in AD onset.

### 5.1. PGD_2_ and 15d-PGD_2_ in AD

Prostaglandin D_2_ (PGD_2_, (Z)-7-[(1R,2R,5S)-5-hydroxy-2-[(E,3S)-3-hydroxyoct-1-enyl]-3-oxocyclopentyl]hept-5-enoic acid) has been shown to be the most abundant eicosanoid in the brain [51,52] and also the most elevated under pathological conditions [53]. PGD_2_ is synthesized from PGH_2_ through the action of hematopoietic prostaglandin D synthase (HPGDS) and the lipocalin-type prostaglandin D synthase (PTGDS, L-PGDS), the latter shown to be one of the more abundant proteins in the brain [54]. There are two known human receptors for this prostanoid, DP_1_ and DP_2_ (CRTH2). HPGDS and both receptors are reported to be expressed in microglia and astrocytes [55]. Both receptors are coupled to G-proteins and upon binding to PGD_2_ or its J_2_ series metabolic products, are reported to have opposite effects on [cAMP] where activation of DP_1_ increases [cAMP] and activation of DP_2_ decreases [cAMP] while increasing intracellular [Ca^2+^] [56]. At physiological concentrations of PGD_2_, activation of DP_1_ receptors have been shown to rescue neurons from glutamate toxicity in cultured rat hippocampal neurons and in organotypic slices [53]. In contrast, activation of the DP_2_ receptor resulted in neuronal loss. Further, stimulation of DP_2_ in Th2 cells preferentially induced the production of inflammatory cytokines [48,57]. It was also established that activated astrocytes express inflammatory cytokines [58]. With this in mind it is not unreasonable that activation of DP_2_ receptors in astrocytes or Th2 cells that have crossed the blood brain barrier (BBB) [59] may release inflammatory cytokines that result in neuronal loss.

Both HPGDS and the DP_1_ receptor have been shown to be upregulated in microglia and reactive astrocytes within senile plaques obtained from human AD brain and in a mouse model of Alzheimer’s disease [60]. The upregulation of each increased in step with Aβ deposition until the plaques reached the “burned out” stage where expression was diminished. Based on the known function of the DP_1_ receptor in neuron rescue, its increased expression is likely a response to the toxic effect of Aβ production in early stages of AD. In all, these results suggest that once plaques begin to form, microglia and astrocytes become activated and produce cytokines that cause further neuronal injury. The involvement of DP_2_ receptors in AD has yet to be elucidated. However, DP_2_ involvement in neuronal degradation is well documented [61]. For example, inhibition of DP_2_ receptors in DP_1_-null twitcher mice resulted in a reduction of astrogliosis, demyelination, and oligodendroglial apoptosis [55]. Clearly, DP_2_ stimulation exacerbates demyelination and enhances inflammation via astrogliosis.

PGD_2_ has been shown to spontaneously dehydrate to either 15-deoxy-prostaglandin D_2_ (15d-PGD_2_; (Z)-7-[(1R,2S,5Z)-2-hydroxy-5-[(E)-oct-2-enylidene]cyclopent-3-en-1-yl]hept-5-enoic acid) or prostaglandin J_2_ (PGJ_2_) with an overall brain half-life of ≈1.1 min [62]. 15d-PGD_2_ has been established as a potent activator of the DP_2_ receptor in eosinophils, but does not activate the DP_1_ receptor [63]. The EC_50_ for initiating eosinophil shape change and chemotaxis (≈ 2.2 nM) by 15d-PGD_2_ was found to be similar to that obtained for that initiated by PGD_2_ on DP_2_ receptors expressed in HEK293 cells (1.6 mM) [56] and thus could potentially be involved in neuronal degradation. However, the data reported to date are mixed. Toll-like protein receptors (TLR) are expressed on the surface of immune cells and act as the first line of defense where they recognize various inflammatory stimuli and initiate immune cells to release an inflammatory response. It has been shown that microglia and astrocytes pre-treated with 15d-PGD_2_ or PGD_2_ and then stimulated with the inflammatory lipopolysaccharide (LPS) produced significantly less surface expressed TLR2 than cells that were not pretreated with 15d-PGD_2_ or PGD_2_ [64]. This reduction in inflammatory response suggests that a neural protective effect is more in keeping with DP_1_ stimulation than DP_2_ stimulation.

### 5.2. PGJ_2_, 15d-PGJ_2_, and Δ^12^-PGJ_2_ in AD

The J_2_ series is formed by the spontaneous non-enzymatic dehydration of PGD_2_ to prostaglandin J_2_ (PGJ_2_, (Z)-7-[(1S,5R)-5-[(E,3S)-3-hydroxyoct-1-enyl]-4-oxocyclopent-2-en-1-yl]hept-5-enoic acid). PGJ_2_ is quite unstable itself and spontaneously and non-enzymatically dehydrates to 15d-PGJ_2_ ((Z)-7-[(1S,5E)-5-[(E)-oct-2-enylidene]-4-oxocyclopent-2-en-1-yl]hept-5-enoic acid) or in the human serum albumin (HSA) catalyzed rearrangement to Δ^12^-PGJ_2_ ((Z)-7-[(1S,5E)-5-[(3S)-3-hydroxyoctylidene]-4-oxocyclopent-2-en-1-yl]hept-5-enoic acid). Biological half-lives of each member of the series appear short, but specific values are unreported.

The J_2_ series of prostaglandins (e.g., PGJ_2_ and Δ^12^-PGJ_2_) have proved to be some of the most neurotoxic compounds within the PGD_2_ degradation pathway [65,66]. These prostaglandins have very short reported half-lives [67]. There are no known J_2_ series specific receptors reported to date. Although the EC_50_ reported for both DP_1_ and DP_2_ receptors are close to that observed for PGD_2_ [56,68] there are no reports of DP receptor involvement in the biological activity of PGJ_2_. Both 15d-PGJ_2_ and Δ^12^-PGJ_2_ have been shown to have similar EC_50_s for DP_2_ receptors, but much poorer EC_50_s for DP_1_ [56,68]. The only the known biological action demonstrated for 15d-PGJ_2_ was as a DP_2_ agonist [63]. The effect of J_2_ series prostaglandins on neurons is complex and appears to act mainly through receptor-independent mechanisms. The reactivity of protein thiols with the α,β-unsaturated carbonyl center on the cyclopentanone ring of J_2_ series is central to their activity and has been shown to be involved in neuron decline. The receptor-independent action of the J_2_ series on intracellular proteins necessarily requires reuptake, requiring a mechanism for cross-membrane transport. PGD_2_ is observed to be a substrate of prostaglandin transporters (PGTs) [69] which are known to be present in the brain [70]. Its transport and subsequent dehydration to PGJ_2_ metabolites is likely involved in the intracellular action. Prostaglandins, including PGD_2_ and PGJ_2_ have also been shown to be transferred from cell to cell via exosomes [71].

Upon treatment of human neuroblastoma SK-N-SH cells with PGJ_2_, an initial time-dependent accumulation of ubiquitinated proteins (Ub) was observed [67]. This was followed by activation of caspase cleavage of tau at D141, leading to the production of Δtau, an early event in AD pathology [72]. These events were followed later by cellular apoptosis. Both PGJ_2_ and PGD_2_ treatment of rat E18 cortical neuronal cells were also shown to generate Δtau. However, treatment of rat primary neuron-enriched cultures with DP_1_ and DP_2_ antagonists did not affect the accumulation of Ub proteins [66]. This observation, the known neuroprotective effect of PGD_2_ through the DP_2_ receptor, and the rapid non-enzymatic conversion of PGD_2_ to PGJ_2_ and its metabolites indicate that the enhanced Ub accumulation following treatment with PGD_2_ is due to its metabolic byproducts.

The mechanism for the accumulation of Ub proteins involved in AD has been ascribed in part to the reaction of PGJ_2_ and Δ^12^-PGJ_2_ with a thiol on ubiquitin C-terminal hydrolase L1 (Uch-L1) [65], a major neurological protein that functions to remove ubiquitin from misfolded proteins that need to be directed to the proteasome pathway [73]. Inhibition of this enzyme has been shown to lead to an increase in Aβ_42_, which in turn downregulates Uch-L1 further [74]. It has been shown that inhibition of Uch-L1 is facilitated by reaction of a single thiol with the α,β-unsaturated carbonyl center on the cyclopentanone ring of PGJ_2_ series prostaglandins, leading to denaturation and aggregation of the enzyme and loss of activity [75]. As noted above, PGD_2_ is known to spontaneously dehydrate to either 15d-PGD_2_ or PGJ_2_. It has been shown that neither PGD_2_ nor 15d-PGD_2_ react with Uch-L1 and that the α,β-unsaturated carbonyl ring containing PGJ_2_ does. Thus, dehydration of PGD_2_ to 15d-PGD_2_ rather than PGJ_2_ passively helps to reduce Aβ_42_ accumulation. The conditions favoring one path over the other are currently unknown.

In contrast to the pro-inflammatory actions of the J_2_ series, 15d-PGJ_2_ exhibits anti-inflammatory properties. 15d-PGJ_2_ is a natural ligand for peroxisome proliferator-activated receptor γ (PPARγ). PPARγ is known to participate in various biological functions including lipid homeostasis, glucose metabolism, macrophage functions, and anti-inflammatory responses [76]. It is a nuclear receptor and serves to activate a number of genes. Ischemia-reperfusion injured rat brain showed increased production of 15d-PGJ_2_ and PPARγ. Intraventricular infusion of 15d-PGJ_2_ into this rat model has been shown to suppress ischemic brain infarction, neuronal apoptosis necrosis in a PPARγ dependent manner [77]. PPARγ has been found to be present in elevated levels in the brains of individuals with AD as well as in other CNS disorders [78,79]. In animal models of AD, treatment with PPARγ agonists reduced amyloid plaque burden, reduced inflammation, and reduced AD behavioral impairment [78]. Similar results were observed in human AD patients and showed improved memory impairments for those with mild-to-moderate AD [80]. 15d-PGJ_2_ has also been shown to suppress several genes involved in neural inflammation and AD including inducible NO synthase and tumor necrosis factor α (TNFα) in a PPARγ-dependent manner [81,82,83,84]. 15d-PDJ_2_ also reduced inflammation by directly inhibiting NF-κB gene expression thereby suppressing COX-2 expression and concomitant prostaglandin synthesis [81]. The PPARγ-independent inhibition appeared to involve the Michael-type addition of the α,β-unsaturated carbonyl center on the cyclopentanone ring to a specific cysteine residue on the NF-kB activator IκB kinase (IKK) resulting in inhibition and by directly inhibiting binding of NF-kB to DNA [76].

### 5.3. PGI_2_ in AD

Prostaglandin I_2_ (PGI_2_, prostacyclin, (5Z)-5-[(3aR,4R,5R,6aS)-5-hydroxy-4-[(E,3S)-3-hydroxyoct-1-enyl]-3,3a,4,5,6,6a-hexahydrocyclopenta[b]furan-2-ylidene]pentanoic acid) is synthesized from PGH_2_ by prostacyclin synthase (PTGIS) and serves as a vasodilator, platelet inhibitor, and is involved in neuron survival [85,86]. PTGIS has been observed to be localized in blood vessels throughout the brain as well as in microglia, and oligodentrocytes [87]. The strongest expression was observed in large principal neurons, pyramidal cells of the cortex and hippocampus as well as the purkinje cells of the cerebellum. PGI_2_ is quite unstable at physiological pH with a measured half-life of 2–5 min, forming biologically inactive 6-keto-prostaglandin F1α (6-keto-PGF1α) [86]. The PGI_2_ receptor (IP) is a G-protein coupled receptor having very low cross-reactivity with other prostanoids [88] and signals through either cAMP or IP_3_/Ca^2+^ mechanisms depending on the G-protein it couples with [89]. In situ hybridization in murine brain revealed that IP receptor mRNA was expressed in about 40% of the neurons in the dorsal root ganglion, but no labeling was found in the glia [90]. Radiolabeling with the stable PGI_2_/IP agonist [^3^H]iloprost showed high binding in the lower brainstem of rat brain and moderate binding in the thalamus, cerebral cortex, and hippocampus.

The PGI_2_/IP signaling pathway plays a crucial role in the metabolism of APP. In a murine model, iloprost stimulation of IP receptors stimulated production of APP and the metalloproteinase ADAM10, resulting in an increased production of the neuroprotective soluble APPα (sAPPα) [91]. In addition, iloprost stimulation of IP receptors in human brain microvascular endothelial cells resulted in upregulation of PPARγ, a protein known to reduce inflammation and amyloid plaque burden [91]. PGI_2_ was shown to suppress the expression of interferon γ (INFγ), a cytokine associated with inflammation and known to accelerate Aβ_42_ aggregation [92]. PGI_2_ was also found to suppress the activation of astrocytes, thus reducing their expression of inflammatory cytokines. In keeping with PGI_2_ neuroprotection, PGI_2_ has also been observed to protect mouse brain pericytes from lysophosphatidylcholine (LPV)-mediated apoptosis [93] thereby preventing BBB disruption, a known characteristic of AD [94], and AD-like neurodegeneration in mice [95].

### 5.4. PGE_2_ in AD

Prostaglandin E_2_ (PGE_2_, (Z)-7-[(1R,2R,3R)-3-hydroxy-2-[(E,3S)-3-hydroxyoct-1-enyl]-5-oxocyclopentyl]hept-5-enoic acid) is synthesized from PGH_2_ by microsomal PGE_2_ synthase-1 (PTGES, mPGES-1), microsomal PGE_2_ synthase-2 (PTGES2, mPGES-2), and cytoplasmic PGES-3 (PTGES3, cPGES), each having a different structure and several having different cellular locations [96,97]. Two of the known isoforms are glutathione requiring (PTGES, PTGES3) and one is glutathione independent (PTGES2). In normal human brains PTGES2 and PTGES3 are highly expressed in the mitochondria and nucleoplasm/cytoplasm respectively and PTGES shows very low expression and is found in the ER. Tissue samples from human middle frontal gyrus have been evaluated for the presence of specific prostaglandin E_2_ synthases. PTGES has been found in neurons, microglia, and endothelial cells of the brain in both AD and controls and elevated in AD tissue, particularly in the pyramidal neurons [98]. Immunostaining revealed that PTGES co-localizes with Aβ in microglia-shaped cells in both AD and controls. PTGES2 has been found in neurons, activated microglia, and endothelium, but not resting microglia, astrocytes of smooth muscle cells in both AD and controls, and found to be elevated in AD pyramidal neurons [99]. The expression pattern for PTGES3 has been shown to be similar to PTGES2 in both AD patients and controls, however, its expression was found to be decreased in the pyramidal cells of AD patients [100].

PGE_2_ is quite stable for a prostaglandin with a reported half-life of about 8.8 h and is metabolized to primarily 13,14-dihydro-15-keto-PGE_2_ and smaller amounts of prostaglandin B_2_ (PGB_2_). There are four distinct subtypes (EP_1_–EP_4_) and nine known PGE_2_ receptors, each of which couple to various G proteins, stimulating a variety of signal pathways [101].

The EP_1_ receptor is highly expressed in the cerebellum, the Purkinje neurons in particular [102] and is known to couple with the G_q_ protein to signal through the IP_3_/Ca^2+^ signaling pathway [103]. Although EP_1_ is also expressed in microglia, its presence was found to be undetectable in microglia after middle cerebral artery occlusion (MCA) [104]. Stimulation of the EP_1_ receptor resulted in an increase in [Ca^2+^] leading to suppression of the pro-survival protein kinase AKT, ultimately resulting in neuronal cell death in mice following an ischemia/reperfusion insult [105]. EP_1_^−/−^ mice and those treated with the EP_1_ antagonist SC50189 showed significantly less neural damage after being subjected to ischemia/reperfusion. These results suggest that EP_1_ stimulation reduces neural viability. In support of this, another study revealed that fewer amyloid plaques were found in EP_1_^−/−^ AD mice compared to AD mice [106]. Further, the EP_1_^−/−^ mice were shown to be more resistant to Aβ neural toxicity than wild type mice. These results show that EP_1_ stimulation is clearly involved in Aβ production and increased susceptibility to Aβ toxicity. In addition, treatment of human neuroblastoma cell line MC165 with EP_1_ antagonist SC51089 reduced Aβ neurotoxicity by 50%; inhibition of the IP_3_/Ca^2+^ signaling pathway produced similar results [107].

In contrast to EP_1_, the EP_2_ receptor involvement in AD is microglial in nature. EP_2_ receptors are known to couple to both G_s_ and G_q_ proteins, leading to cAMP/PKA or IP_3_/Ca^2+^/AKT signaling respectively. The fact that microglial signaling via EP_2_ initiated PKA signaling [108] indicates that the G_s_/cAMP signaling pathway is at work in microglia. In AD model mice, microglial EP_2_ expression and Aβ_42_ accumulation increased with aging [109]. In the same study the EP_2_^−/−^ AD model showed an increase in microglial chemotaxis and Aβ clearance, a suppression of toxic inflammation, and an increase in the insulin-like growth factor 1 (IGF1) signaling, known for its involvement in neuron survival. EP_2_ receptors have also been shown to be involved in the increase in lipid peroxidation and associated Aβ_40_ and Aβ_41_ plaque load in aged mice [110]. Microglial phagocytosis of Aβ is essential to a reduction of Aβ plaque load and progression of AD. Two studies have shown that stimulation of EP_2_ reduced microglial phagocytosis. Stimulation of murine N9 microglia cells in culture with either PGE_2_ or PKA agonists reduced the ability of these cells to phagocytose, whereas treatment with the EP_2_ antagonist AH6809 restored the ability to phagocytose [108]. In addition, treatment of human hippocampal sections from patients who died with AD with microglia isolated from EP^−/−^ mice reduced the Aβ_40_, Aβ_41_, and aggregated Aβ_42_ load, whereas microglia from WT mice had no effect [111].

The EP_3_ receptor involvement in AD is the least well defined of all EP receptors. This is likely due to the fact that there are at least six known human isoforms of EP_3_ identified, each of which has a particular set of G-proteins that it binds to and thus signaling pathways vary widely [112]. Isoforms EP3-I, EP3-II, EP3-III, and EP3-IV are all found in brain tissue, whereas EP3-V and EP3-VI appear to be restricted to uterine tissue. To date there are no reports on the involvement of specific EP_3_ isoforms in AD, but a number involving a generic reference to the EP_3_ receptor. It is established that EP_3_ receptor expression is primarily in neurons [113,114] and couples with G_i_ proteins, leading to a decrease in cAMP as the signaling mechanism. However, under neurodegenerative conditions, expression of EP_3_ in rat microglia was significantly enhanced [113]. Similar results were obtained for EP_3_ expression in post-mortem human temporal cortexes. Here, basal EP_3_ levels were also found in control samples, but expression in AD samples were found to be expanded to include glial cells, in particular, those areas surrounding amyloid plaques [115]. Further, protein levels for EP_3_ increased from controls to samples from mildly cognitively impaired patients (MCI) to AD states, indicating that EP_3_ is directly involved in the progression of AD. More specifically, EP_3_ signaling has been shown to induce pro-inflammatory gene induction, cytokine production, lipid peroxidation, and generation of Aβ peptides in APPswe-PS1 DE9 AD model mice. A second study using the same mouse AD model revealed that PGE_2_ levels rise markedly with aging and PGE_2_-EP_3_ signaling impaired presynaptic LTP [116].

EP_4_ signaling contrasts markedly with other EP receptors with its beneficial anti-inflammatory and AD resolving effects. Its involvement in AD is primarily via microglia. EP_4_ is known to couple to G_s_, G_q_, and G_i_ proteins leading to and cAMP/PKA signaling, IP_3_/Ca^2+^/AKT signaling, or a decrease in cAMP respectively [89,117]. Treatment of murine microglial-like BV-2 cells with the specific EP_4_ agonist AE1-329 with and without LPS stimulation significantly increased PKA activity, confirming the cAMP/PKA signaling pathway through G_s_ coupling is at work in microglia [118]. Isolated microglia obtained from EP_4_^+/+^ AD model mice (APPswe-PS1 DE9; EP_4_^+/+^) stimulated with EP_4_ specific agonist AE1-329 showed a suppressed oxidative and cytokine/chemokine response induced by Aβ_42_ [119] while it increased the expression of anti-inflammatory factors. In particular, expression of NF-kB, IRF1, and IRF7 networks were suppressed and a marked increase in the anti-inflammatory cytokine IL-10 was observed [118]. Further, AE1-329 stimulation of EP_4_ also induced phagocytosis of Aβ_42_. Other studies have shown that the phosphorylation of tau required for deposition is not facilitated by EP_4_, but instead by EP_1–3_ [120]. Stimulation of EP_4_ has also been shown to indirectly ameliorate AD damage by reducing matrix metalloproteinase (MMO)-3/-9 expressions that would otherwise compromise the integrity of the BBB by cleaving basement membrane proteins of the neurovascular unit and tight junction proteins [121].

The function of EP_4_ in brain tissue is juxtaposed to that of EP_1–3_, suggesting that its normal role involves neural homeostasis. However, during the advance of normal to MCI to AD, the homeostasis is clearly failing. In patient samples, EP_4_ expression has been shown to decrease when progressing from normal to MCI to AD, particularly in neurons and glia while EP_3_ expression increased [119]. Further, with aging or Aβ_42_ accumulation, microglial EP_2_ has been shown to be upregulated, increasing pro-inflammatory gene expression, suppressing beneficial chemokine production, chemotaxis [109], and phagocytosis [119]; at the same time EP_4_ expression was reduced. One proposed mechanism for the reduction in EP_4_ expression involves the co-internalization of EP_4_ and the PS-1 subunit of γ-secretase into endosomes. The fact that an increase in EP_2_ expression leads to an increase in both Aβ and γ-secretase production suggests that EP_2_ expression drives EP_4_ internalization. Although this observation provides an explanation for the reduction of the surface expression of EP_4_ it also presents an unresolved quandary. Since both EP_4_ and EP_2_ are expressed on microglia and both signal through a cAMP/PKA signaling pathway, how do the opposing functions operate?

### 5.5. TXA_2_ in AD

Thromboxane A_2_ (TXA_2_, (Z)-7-[(1S,3R,4S,5S)-3-[(E,3S)-3-hydroxyoct-1-enyl]-2,6-dioxabicyclo[3.1.1]heptan-4-yl]hept-5-enoic acid) is a potent vasoconstrictor and platelet activator [122], quite the opposite of PGI_2_. Thromboxane A_2_ (TXA2) is synthesized from PGH_2_ by thromboxane A synthase (TBXAS1), a monomeric, heme-requiring, ER transmembrane protein and a member of the cytochrome P450 superfamily. TBXAS1 is expressed in most tissues, except for the skin, and in particularly high amounts in the spleen, brain, bone, immune system, and lungs. Immunostaining revealed significant expression in glial cells, macrophages, platelets, and lung fibroblasts. TXA_2_ is known to be quite unstable with a reported half-life of 30 s under physiological conditions and hydrolyzes to the stable, biologically inactive thromboxane B_2_ (TXB_2_) [123].

There are two isoforms of the TXA_2_ receptor (TBXA2R), TPα and TPβ, which differ in the sequence and length of the C-terminal tail that determines the G-protein coupling and hence signaling pathway [124]. Stimulation of both receptors has been shown to lead to an increase in IP_3_ and Ca^2+^, indicating a G_q_ mediated pathway for both receptor isoforms. However, their effect on cAMP production was shown to be opposing, where TPα increases cAMP and TPβ inhibits cAMP, suggesting additional coupling to G_s_ and G_i_ proteins respectively. TP receptors also couple with G_13_ proteins, shown to result in RhoGEF activation [125], a signaling pathway known to be involved in a number of neurological disorders [126]. The TP isomer preference for G_13_, if any, has yet to be determined.

TBXA2R in brain tissue is found in neurons [127] as well as activated microglia and macrophages [128]. Several reports have shown that stimulation of TP receptors results in an increase in the expression of APP and deposition of Aβ [129,130]. Further, treatment of a human QBI293 cell line expressing hAPP and human TP receptor (hTP) with siRNAs for G_q_, G_12_, and G_13_ revealed that TP receptor stimulation led to APP expression proceeding through G_q_ coupling. As noted above, activation of microglia/macrophages contributes to the inflammatory response and oxidative stress associated with AD. In a murine ischemic stroke-induced model, TP receptor expression was significantly increased in microglia/macrophages localized in the damaged area [128]. Administration of the TP antagonist SQ29548 to these model mice inhibited microglia/macrophage activation and significantly reduced secretion of inflammatory cytokines (e.g., IL-1β, IL-6, TNFα), indicating that stimulation of TP receptors facilitated the activation and cytokine release. Further, treatment of 1321N1 astrocytoma cells with the TP receptor agonist U46619 increased the production of the pro-inflammatory IL-6 and the cyclic AMP-response element protein (CREB) [131], supporting TP-mediated microglia activation. The fact that CREB induction requires an increase in cAMP suggests that the TPα isomer is involved.

TP receptors in platelets also contribute to the pathogenesis of AD. Cerebral amyloid angiopathy (CAA) is characterized by Aβ deposits in the walls of cerebral vessels and was found in 98% of AD patients, and ultimately led to degeneration of vessel wall components and a reduction in cerebral blood flow, thus aggravating cognitive decline. It has been shown that more than 90% of circulating Aβ is produced by activated platelets. Aβ itself has been shown to activate platelets further which is augmented with TP stimulation leading to overproduction of Aβ and progression of CAA [132]. Recently, it has been proposed that circulating Aβ produced by platelets move from blood to brain tissue and serve as “seeds” to initiate Aβ aggregation of brain-produced Aβ [118].

### 5.6. Isoprostanes in AD

Under conditions of oxidative stress, reactive oxygen species (ROS) are produced at higher than normal levels and can facilitate the peroxidation of PUFAS, including AA. Isoprostanes are prostaglandin-like molecules produced by non-enzymatic peroxidation of AA. It is well established that oxidative stress plays a role in AD pathogenesis. One class of products produced by oxidative stress is the F2-isoprostanes obtained from AA, the concentration of which have been shown to be increased in brain and CSF of AD patients [133,134]. In particular, isoprostane-F_2α_-III (iPF_2α_-III, 9α,11α,15S-trihydroxy-(8β)-prosta-5Z,13E-dien-1-oic acid) and isoprostane F_2α_-VI (iPF_2α_-VI, (8β)-5,9α,11α-trihydroxy-prosta-6E,14Z-dien-1-oic acid) were found to be higher in AD post-mortem human brains than controls [133]. This raises the question as to whether isoprostanes are merely markers of oxidative stress or actually participate in AD pathology. There is significant amount of literature supporting the latter. Numerous reports have indicated that isoprostanes bind to and activate TP receptors (e.g., [135,136]) and thus can be involved in AD pathogenesis as described above. Direct evidence for isoprostane involvement in AD was obtained through experiments where iPF_2α_-III was injected into Tg2576 AD model mice [137]. The iPF_2α_-III increased both steady-state APP levels and the concentrations of Aβ cleavage products. These increases were inhibited if the mice were pre-treated with a TP receptor antagonist, thus suggesting that TP receptor signaling is involved. A second study monitored urinary, plasma, and brain homogenate 8,12-iso-iPF_2α_-VI from Tg2576 mice and found that concentrations were higher in Tg2576 mice than WT as early as eight months of age [138]. By 12 months of age, a “surge” of Aβ_40_ and Aβ_42_ levels occurred and Aβ aggregates were observed in Tg2576 mouse brains and not WT brains. These results suggest that the lipid oxidative events precede plaque formation in AD.

### 5.7. PGF_2α_ in AD

Prostaglandin F_2α_ (PGF_2α_, (Z)-7-[(1R,2R,3R,5S)-3,5-dihydroxy-2-[(E,3S)-3-hydroxyoct-1-enyl]cyclopentyl]hept-5-enoic acid) is one of the more abundant prostanoids in the brain and spinal cord [139] and is also found in many other tissues. It has a long half-life (15.0 ± 8.2 h) compared to other prostaglandins and is metabolized to 15-keto-PGF_2α_ and then to 13,14-dihydro-15-keto PGF_2α_ enzymatically. The functions are wide ranging and context dependent. PGF_2α_ is involved in inflammation as well as smooth muscle contraction, renal function, and blood pressure to name a few [140]. The stereoisomer 9α,11β-PGF_2_ is thought to have similar functions [141]. PGF_2α_ is synthesized through the action of prostaglandin F synthases. Prostaglandin F synthase represents a collection of enzymes that ultimately produce PGF_2α_ or the stereoisomer 9α,11β-PGF_2_. These enzymes are all produced in the brain and fall into one of three different structural classes: (1) aldo-keto reductase superfamily (AKR1B1, AKRC3), (2) the thioredoxin-like superfamily (FAM213B), and (3) the short-chain dehydrogenases/reductases (SDR) family (CBR1) [142,143]. Substrates for PGF_2α_ production are either PGH_2_ or PGE_2_ depending on the enzyme, whereas 9α,11b-PGF_2_ is produced only from PGD_2_. The properties of these and other NADH/NADPH-dependent reductases have been reviewed [144].

There are seven known human isoforms of the PGF_2α_ receptor (FP), each of which differs in the length and sequence of the C-terminal tail. This leaves open the possibility that signaling might occur through a variety of G proteins. However, only coupling to G_q_ and Rho have been observed, leading to the activation of the IP_3_/Ca^2+^ signaling pathway and cytoskeletal changes respectively [145,146,147,148]. PTGFR is the most promiscuous of the prostanoid receptors, exhibiting measurable binding to a number of prostanoids. A few relative pK_i_ values were reported as follows: PGF_2α_ (7.9–8.5) > PGD_2_ (7.3–7.7) > PGE_2_ (7.0) > PGI_2_, TXBA_2_ where only PGD_2_ and PGE_2_ are likely alternate agonists under physiological conditions.

There are few reports describing the involvement of PGF_2α_ in AD and much of it provides only an indirect relationship. Immunohistochemical staining of post-mortem hippocampal tissue obtained from AD and control brains showed significantly elevated levels of the PGF_2α_ metabolite 13,14-dihydro-15-keto PGF_2α_ in AD tissue than in aged controls, implicating high PGF_2α_ concentrations at some time in the development of AD [148]. Treatment of human BV2 microglial cells with t0901317, a known liver X receptors/retinoid X receptor α (LXR/RXR) agonist, reduced the transcription of the inflammatory cytokines IL-6 and TNFα, following an inflammatory challenge with LPS or Aβ and promoted Aβ clearance in the latter challenge [149]. Addition of PGF_2α_ to the treatment regime antagonized t0901317 in a dose dependent manner suggesting that PGF_2α_ might be involved in the progression of AD.

Most of the reports focus on the action of PGF_2α_ in damaged brain tissue, in particular, oxidative damage as is found in early AD. FP^−/−^ mice subjected to ischemic-reperfusion (IR) injury showed less neurological deficit (25.3% less) and smaller infarct volumes (34.4% less) than identically treated WT mice. Similarly, mice treated with the FP antagonist AL-8810 after 48 h of permanent middle cerebral artery occlusion (pMCAO) showed less neurological dysfunction (35% less) and smaller infarct volumes (36.4%) than did saline-treated controls [134]. FP^−/−^ mice also had improved outcomes after pMCAO. In a related study, FP^−/−^ mice subjected to controlled cortical impact (CCI) showed significantly less hippocampal swelling and accumulation of astrocytic and microglial markers than identically treated WT controls [150]. Following CCI, post-treatment of WT mice with AL-8810 significantly reduced hippocampal swelling compared to untreated controls. Repeated treatments with AL-8810 had no effect, indicating that the FP receptor is only involved in early events in brain damage. These studies consistently indicate that stimulation of FP exacerbates neuronal damage following a brain insult and suggests that the FP is involved in early events following the insult and possible in early events in AD.

It is well established that treatment of neuron cultures with Aβ induces cell death. It has been shown that treatment of rat cortical neuron cultures with Aβ and 10 µM PGD_2_ or PGJ_2_ reduces neuron survival by 70% and 79% respectively compared to Aβ only treated cultures [151]. Repeating the experiment with 10 µM PGF_2α_ or 9α, 11β-PGF_2α_ had no effect on cell survivability. This study is consistent with PGF_2α_ or its isomer not being involved in Aβ toxicity. In light of the previous results, the FP receptor’s neurotoxic activity appears to be involved in early events leading to AD and prior to Aβ release.

## 6. Lipoxygenases, and their Products and Receptors in AD

### 6.1. HETEs in AD

**Figure 2:**
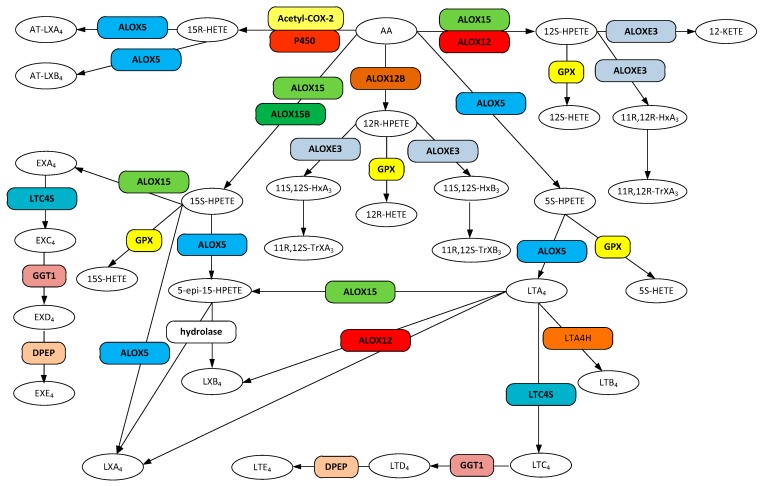
Metabolic pathways for hydroxyeicosatetraenoic acid (HETEs), lipoxin (LX), AT-lipoxin (AT-LX), hepoxilin, leukotriene (LT), and eoxin (EX) biosynthesis from arachidonic acid. Gene designations are given for all but the COX participating enzymes (rounded boxes) and accepted acronyms given for metabolites (ovals).

The biosynthesis of hydroxyeicosatetraenoic acids (HETE), lipoxins, leukotriene A_4_, and hepoxilins are accomplished by a group of structurally similar enzymes of the lipoxygenase family (Figure 2). Lipoxygenases represent a non-heme, iron-requiring class of enzymes that oxygenate an array of PUFA substrates; see review by Biringer [152]. The HETE family of molecules and their unstable precursors, the hydroperoxyeicosatetraenoic acids (HPETEs), are notable as precursors for the leukotriene family, lipoxin family, trioxilin family, and eoxin family of metabolites, as well serving as signaling molecules in their own right [153].

The mammalian lipoxygenase ALOX15 (12/15-LOX, 15-LOX, 15-LOX-1, 12/15LO) is a monomeric cytosolic protein that becomes membrane associated in the presence of calcium [154,155]. Both substrate and product specificity are diverse (Figure 2). ALOX15 converts arachidonic acid into 12(S)-hydroperoxyeicosatetraenoic acid (12S-HPETE, (5Z,8Z,10E,12S,14Z)-12-hydroperoxyicosa-5,8,10,14-tetraenoic acid) and 15(S)-hydroperoxyeicosatetraenoic acid (15S-HPETE, (5Z,8Z,11Z,13E,15S)-15-hydroperoxyicosa-5,8,11,13-tetraenoic acid) in a ratio of 1:9 [156,157] that are readily converted to the corresponding hydroeicosatetraenoic acids by the action of the ubiquitous glutathione peroxidases (GPX) [158]. In addition, ALOX15 not only oxygenates free fatty acids, but also membrane phospholipids when in the membrane bound state [154] and generates 15S-hydroxyeicosatetraenoic acid (15-HETE, (5Z,8Z,11Z,13E)-15-hydroxyicosa-5,8,11,13-tetraenoic acid) conjugated to phosphatidylethanolamine, an intracellular signaling molecule [159].

ALOX15 is one of the most abundant lipoxygenase isoforms in the CNS and is found in both neurons and glial cells with 12S-hydroxyeicosatetraenoic acid (12-HETE, (5Z,8Z,10E,14Z)-12-hydroxyicosa-5,8,10,14-tetraenoic acid) and 15-HETE as the major products [160]. Analysis of post-mortem brains from AD patients and controls revealed that ALOX15 is significantly upregulated in AD patients and the concentrations of both 12-HETE and 15-HETE are markedly elevated in the frontal cortex and temporal cortex, but not the cerebellum of AD brains [160]. Human ALOX15 expressed in CHO and N2A cell lines treated with selective ALOX15 inhibitors reduced Aβ formation in a dose dependent manner [161,162]. Further, β-secretase (BACE) and secreted APPβ (sAPPβ) were also reduced whereas soluble APPα and the ADAM10 protein were unchanged, indicating that either or both 12-HETE and 15-HETE modulates the BACE proteolytic cascade. A Tg2576 murine AD model overexpressing ALOX15 showed elevated levels of phosphorylated tau [163]. Further examination revealed that this process proceeded through the cdk5 kinase pathway and was independent of the effect on Aβ. A global lipidomic analysis of brain tissue from Tg2576 mice stably expressing human APP and tau protein revealed that the concentration of 12-HETE nearly doubled from four months of age to 10 months of age and was then reduced to nearly the four month level at 15 months when compared to WT mice of the same ages [164]. In contrast, 15-HETE concentrations decreased by 10% over the 15-month lifetime. AD progression was also monitored as a function of Aβ and tau production. Here it was found that Aβ accumulation begins at 10 months while the levels of soluble tau remained constant. These results are a clear indication that 12-HETE is involved in the early stages of AD. The continual decrease in 15-HETE with age and AD progression is consistent with the potential anti-inflammatory action of this metabolite, as it is a ligand for PPARγ, a known anti-inflammatory receptor [76,165]. The fact that 12-HETE is also a ligand for PPARγ [166] suggests that the concentration increase to the point of Aβ production may be a neuroprotective response that decreases in response to Aβ as its concentration increases.

Arachidonate 5-lipoxygenase (ALOX5, 5-LO, 5-LOX, 5-lipoxygenase) is a member of the lipoxygenase family of proteins. ALOX5 is expressed primarily in cells that are involved in regulating inflammation as well as in neurons and glial cells [167,168]. Although ALOX5 persists in the cytosol, upon an increase in intracellular Ca^2+^, binding of the cation to the PLAT domain of the protein causes it to become associated with the nuclear membrane [169]. ALOX5 catalyzes the conversion of arachidonic acid to 5-hydroperoxyeicosatetraenoic acid (5-HPETE, (6E,8Z,11Z,14Z)-5-hydroperoxyicosa-6,8,11,14-tetraenoic acid) which is then rapidly converted to other products (Figure 2). Release of 5-HPETE to ubiquitous cellular glutathione peroxidases (GPX) results in its reduction to 5-hydroxyeicosatetraenoic acid (5-HETE, (5S,6E,8Z,11Z,14Z)-5-hydroxyicosa-6,8,11,14-tetraenoic acid) [168]. Alternatively, ALOX5 may convert the transient 5-HPETE to leukotriene A_4_ (LTA_4_), the precursor to the synthesis of the other leukotrienes [168]. Additionally, ALOX5 can convert the ALOX15 product 15-HPETE to the epoxide product 5(6)-epoxy-15(S)-hydroxyeicosatetraenoic acid (5-epi-15(S)-HPETE, (5Z,8Z,11Z,13E,15S)-15-hydroperoxyicosa-5,8,11,13-tetraenoic acid), a precursor to the synthesis of lipoxin A_4_ (LXA_4_) and lipoxin B_4_ (LXB_4_) [170,171].

ALOX5 has been shown to be upregulated in post-mortem human AD glial and neuronal cells when compared to controls [172]. ALOX5 was found to be particularly abundant in glial cells with Aβ plaques and vasculature and was also found in tau and amyloid containing neurofibrilliary tangles. In the Tg2576 murine AD model, ALOX5 products increased the formation of Aβ by activating the CREB protein which in turn activated γ-secretase expression [173]. Brain tissue from the same model showed significantly higher amounts of 5-HETE when compared to WT tissue. Treatment of N2A-APPswe cell line, a murine cell line expressing human APP with the AD-prone Swedish mutation, with 5-HETE significantly increased Aβ production and was not associated with APP, BACE1, or ADAM-10 levels [173]. In a similar study, treatment of HEK293 cells stably expressing the C-terminal fragment of human APP (HEK293-C99) with 5-HPETE and leukotriene C_4_ (LTC_4_) induced significant increases in Aβ_40_ [174]. In addition, treatment with 5-HPETE induced a significant increase in γ-secretase production while LTC_4_ treatment led to a milder trend to an increase. More recently it has been shown that ALOX5 products are also involved in the increase of γ-secretase activating protein (GSAP), levels which in turn led to an increase in γ-secretase activity and Aβ production [175]. The effect was indirect through activation of caspase-3 which converts the GSAP precursor to GSAP.

12-HETE and 15-HETE bind to other receptors in addition to the PPARγ binding. Only 12-HETE has been found to have a specific high-affinity membrane receptor, GPR31 (GPBA) [176,177]. Although the relationship between this receptor and AD has yet to be established, this receptor is known to be expressed in the brain [178]. Both 12-HETE and 15-HETE have been shown to bind to the leukotriene B_4_ (LTB_4_) receptor BLT2, albeit with lower efficiency than LTB_4_ [179]. IC_50_ values are reported to be 10 and 50 times higher and EC_50_ values 100 and 500 times that for LBT_4_ respectively, suggesting that the effect exhibited by these HETEs on BLT2 is likely minimal unless leukotriene A_4_ hydrolase (LTA4H) or ALOX5 are downregulated or inhibited.

### 6.2. Leukotrienes and Their Receptors in AD

The leukotriene family of molecules consists of oxygenated products of arachidonic acid of which several are derivatized by glutathione. The molecules are created through the action of ALOX5 on arachidonic acid, resulting in the transient formation of 5S-HPETE which is then converted by ALOX5 to leukotriene A_4_ (LTA_4_, 4-[(2S,3S)-3-[(1E,3E,5Z,8Z)-tetradeca-1,3,5,8-tetraenyl]oxiran-2-yl]butanoic acid). The highly unstable LTA_4_ can be converted by hydrolysis to leukotriene B_4_ (LTB_4_, (5S,6Z,8E,10E,12R,14Z)-5,12-dihydroxyicosa-6,8,10,14-tetraenoic acid).

LTA_4_ is a non-cysteinyl leukotriene produced by ALOX5 from 5-HPETE as described above. Treatment of human neuronal cell lines producing the C-99 fragment of APP (MC65) with LTA_4_ potentiated the Aβ toxicity of the neurons [180]. In contrast, there are no reported receptors for LTA_4_ and the mechanism by which it potentiates toxicity is unknown. However, the highly chemically reactive allylic epoxide renders it quite unstable with an in vitro half-life of less than 3 s at physiological pH and temperature [181] and it is non-enzymatically converted to Δ^6^-trans-LTB_4_ ((5S,6E,8E,10E,12R,14Z)-5,12-dihydroxyicosa-6,8,10,14-tetraenoic acid) and 5,6-diHETE ((8Z,11Z,14Z,17Z)-5,6-dihydroxyicosa-8,11,14,17-tetraenoic acid) [167]. In addition, LTA_4_ is known to be readily converted enzymatically to either LTB_4_ or LTC_4_. Further, LTB_4_ has been reported to be more chemically stable than its precursor LTA_4_ with a considerably longer physiological half-life (25 min) [182]. For these reasons it is reasonable to assume that LTA_4_ merely serves as a substrate to produce products that are responsible for the potentiation of neural toxicity.

LTB_4_ is a non-cysteinyl leukotriene produced by the hydrolysis of LTA_4_ by leukotriene A_4_ hydrolase (LTA4H). The pro-inflammatory LTB_4_ is one of the most potent chemotactic molecules known and induces recruitment and activation of monocytes, neutrophils, and eosinophils [183,184,185]. It is also a potent smooth muscle constrictor and has the ability to increase vascular permeability [182]. Treatment of cultured monocytes with LTB_4_ has been shown to activate MAPK and PI_3_/AKT pathways, and induce overproduction of IL-6, monocyte chemoattractant protein 1 and TNFα, all of which are involved in the inflammatory response.

The production of LTB_4_ was shown to be enhanced in cultured microglia (murine N9) following treatment with Aβ [186]. When COS7 cells transfected with the C-99 protein (see above) were treated with LTB_4_, Aβ_40_ and Aβ_42_ levels were significantly increased over levels in controls [187]. The net effect is a self-proliferating cycle, leading to overproduction of Aβ and eventual deposition. The action of LTB_4_ on Aβ production is indirect. It has been shown that treatment of N2aAbPPswe cells with LTB_4_ increased the cellular concentrations of three of the four protein subunits of the γ-secretase complex [188]. In contrast, ALOX5 increased the concentrations all four of the protein subunits, suggesting that that the fourth subunit APH-1 was induced by another ALOX5 product (e.g., LTD_4_, reference [189]) or that APH-1 levels return to baseline levels within the timeframe of the measurement.

There are two known LTB_4_ G-protein receptors, BLT1 (LTB4R) and BLT2 (LTB4R2). BLT1 is a high affinity form with a reported IC_50_ and EC_50_ for LTB_4_ of about 60 nM and is primarily expressed in leukocytes [190], but also found in spinal neurons [191]. It has a very high specificity for LTB_4_ compared to other eicosanoids. BLT2 is a lower affinity receptor for LTB_4_ with a reported IC_50_ of 100 nM and an EC_50_ similar to that for BLT1. Unlike BLT1, BLT2 is more ubiquitously expressed with a more promiscuous binding specificity. Interestingly, resolvin E1, a potent anti-inflammatory and pro-resolving mediator (see below) and has been shown to selectively bind to BLT1 and antagonize the LTB_4_-induced calcium mobilization and attenuates NF-Kβ activation [192]. Although the relationship of LTB_4_ activation of either receptor to the progression of AD is not yet known, its involvement in associated neuroinflammation is well established.

### 6.3. Cysteinyl Leukotrienes and Their Receptors in AD

The highly unstable LTA_4_ can also be converted to leukotriene C_4_ (LTC_4_, (5S,6R,7E,9E,11Z,14Z)-6-[(2R)-2-[[(4S)-4-amino-4-carboxybutanoyl]amino]-3-(carboxymethylamino)-3-oxopropyl]sulfanyl-5-hydroxyicosa-7,9,11,14-tetraenoic acid) by addition of glutathione. Stepwise hydrolysis of the peptide portion of attached glutathione on LTC_4_ leads to the formation of leukotriene D_4_ (LTD_4_, (5S,6R,7E,9E,11Z,14Z)-6-[(2R)-2-amino-3-(carboxymethylamino)-3-oxopropyl]sulfanyl-5-hydroxyicosa-7,9,11,14-tetraenoic acid) and leukotriene E_4_ (LTE_4_, (5S,6R,7E,9E,11Z,14Z)-6-[(2R)-2-amino-2-carboxyethyl]sulfanyl-5-hydroxyicosa-7,9,11,14-tetraenoic acid) with measured physiological half-lives of 44 min [193], and 22 min respectively [194]. LTE_4_ is quite stable and can be found excreted in the urine. LTC_4_, LTD_4_, and LTE_4_ are known as the cysteinyl-leukotrienes (Cys-LT) and are potent bronchoconstrictors, are known to increase vascular permeability in postcapillary venules, and known to stimulate mucus secretion [184]. They have also been shown to be involved in normal CNS metabolism and numerous CNS disorders [195], including AD [173]. Mammalian leukotriene C_4_ synthase (LTC4S, LTC4 synthase) is a transmembrane protein expressed in a limited number of cell types and is found in mast cells, eosinophils, basophils, and monocytes [196]. The enzyme catalyzes the conjugation of GSH to the unstable LTA_4_ produced by ALOX5, thus producing leukotriene C_4_ (LTC_4_). Subsequent synthesis of LTD_4_ and LTE_4_ occurs through the action of ubiquitous gamma-glutamyl transaminase (GGT1) and dipeptidase (DPEP) respectively. Although ALOX5 is found in neurons and glial cells, an additional source for the Cys-LT, substrate LTA_4_ is also provided by transcellular biosynthesis whereby resident neutrophils produce and transfer the source material [167,197].

There are half a dozen known receptors that bind Cys-LTs. Thus far, only the G-protein coupled CysLT1R receptor has been shown to be directly involved in Aβ pathogenesis. This receptor showed the highest efficiency for LTD_4_ (EC_50_ = 2.5 nM) with much lower efficiencies for LTC_4_ (EC_50_ = 24 nM) and LTE_4_ (EC50 = 240 nM) [198]. A second receptor that may also be involved in AD is the G-protein coupled CysLT2R receptor found in both astrocytes and microglial cells. This receptor showed the highest efficiency for LTC_4_ (67 nM), a slightly lower efficiency for LTD_4_ (104 nM), and very low efficiency for LTE_4_ (2300 nM) [199].

Application of LTD_4_ to cultured neurons has been shown to increase Aβ release and decrease neuronal viability [189]. At low concentrations (20 nM), LTD_4_ caused a significant increase in Aβ generation, whereas at higher concentrations (40 nM) it decreased neuronal viability. Further, the lower concentrations of LTD_4_ also increased the expression of the CysLT1R receptor, increased the activity of β- or γ-secretase, and activated the pro-inflammatory NF-kB pathway. Application of the specific CysLT1R antagonist pranlukast inhibited both Aβ generation and the increase in secretase activity, linking the CysLT1R directly to the response. In keeping with the self-propagating nature of AD, in vivo and in vitro treatment of mouse neurons with Aβ_42_ increased the expression of CysLT1R receptors [200]. Other team players in AD progression are also involved. The CysLT1R receptor is expressed in astrocytes and stimulation rat cultured astrocytes with LTD_4_ resulted in stimulation of astrocyte proliferation, activation of the MAPK pathway, enhanced production of CysLTs, and the release of inflammatory chemokines and cytokines [201]. The effect of LTD_4_ on astrocytes appears to play an autocrine role in the induction of reactive astrogliosis. The involvement of the CysLT2R receptor in microglial activation is controversial. Some reports have shown its upregulation in astrocytes following an inflammatory insult [202] whereas others have reported that it is not expressed in astrocytes at all [201]. Upon microglial activation resulting from inflammation in rat brain, both CysLT1 and CysLT2R were found to be upregulated, however, the subsequent release of inflammatory IL-1β, INF-γ, and TNFα by microglia was inhibited by the selective CysT2R receptor agonist HAMI 3379 [203]. On the other hand, the CysLT1R selective antagonist pranlukast affected only the release of IL-4, indicating that both receptors and both LTC_4_ and LTD_4_ are involved in the inflammatory response, albeit with somewhat different results. The function, if any, of LTE_4_ in AD progression is currently unknown.

### 6.4. Eoxins in AD

In effect, eoxins are the C14,15 oxidized isomers of leukotrienes and are produced from arachidonic acid via ALOX15 to eoxin A_4_ (EXA_4_, (5Z,8Z,10E,12E)-13-[(2S,3S)-3-pentyloxiran-2-yl]trideca-5,8,10,12-tetraenoic acid) and then converted through a linear path to eoxin C_4_ (EXC_4_, (5Z,8Z,10E,12E,14R,15S)-14-[(2R)-2-[[(4S)-4-amino-4-carboxybutanoyl]amino]-3-(carboxymethylamino)-3-oxopropyl]sulfanyl-15-hydroxyicosa-5,8,10,12-tetraenoic acid), eoxin D_4_ (EXD_4_, (5Z,8Z,10E,12E,14R,15S)-14-[(2R)-2-amino-3-(carboxymethylamino)-3-oxopropyl]sulfanyl-15-hydroxyicosa-5,8,10,12-tetraenoic acid) and eoxin E_4_ (EXE_4_, (5Z,8Z,10E,12E,14R,15S)-14-[(2R)-2-amino-2-carboxyethyl]sulfanyl-15-hydroxyicosa-5,8,10,12-tetraenoic acid) by the same enzymes used for the production of leukotrienes (Figure 2). Eoxins are pro-inflammatory metabolites of arachidonic acid and are produced in cells that express significant amounts of ALOX15, for example, human airway epithelial cells, eosinophils, subsets of mast cells, and dendritic cells [204]. Their biological roles have yet to be thoroughly explored, however their role in inflammation suggests they may also be involved in the progression of AD.

## 7. SPMs and Resolution in AD

**Figure 3:**
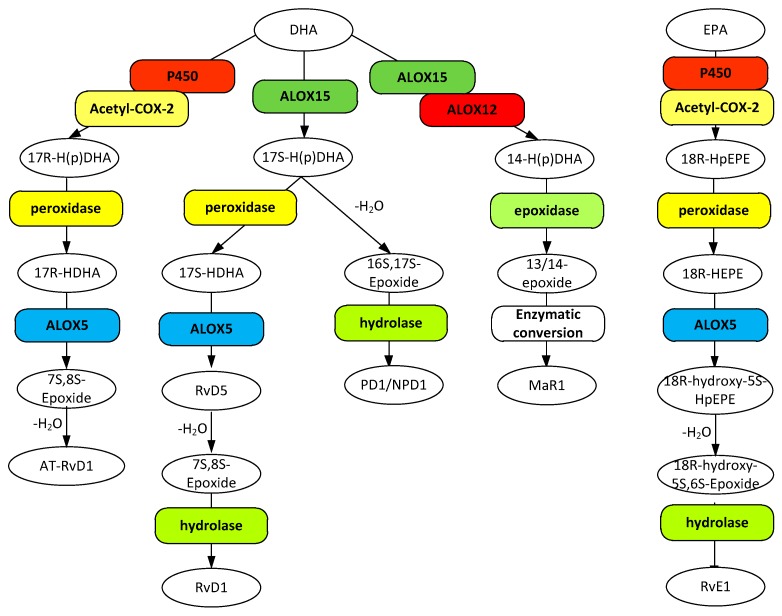
Metabolic pathways for D resolvins (RvD1 and AT-RvD1), protectin-1 (PD1/NPD1), and maresin 1 (MaR1) biosynthesis from docosahexaenoic acid (DHA) and the biosynthesis of resolvin E1 from eicosapentaenoic acid (EPA). Gene designations are given for all but the COX participating enzymes (rounded boxes) and accepted acronyms given for metabolites (ovals).

Resolution of inflammation is essential to the maintenance of tissue homeostasis. Without it, acute inflammation becomes chronic, leading cell death and tissue dysfunction. Central to resolution are specialized pro-resolving mediators (SPM) that serve to prevent uncontrolled inflammation and promote removal of microbes, apoptotic cells and debris to return the tissue to homeostasis (review [205]). SPMs are represented by a series of eicosanoids, namely the lipoxins, resolvin D and E series, protectins, and maresins. The lipoxins are derived from AA (Figure 2) whereas the latter group are synthesized from ω3 fatty acids (Figure 3) [206].

Resolution is key to preventing chronic inflammation that leads to AD progression. Hence, dysfunctional resolution mechanisms are paramount to AD progression. Support for this concept is increasing. For example, post mortem brain tissue and CSF obtained from human AD patients showed significantly lower levels of SPMs than non-AD controls [207]. Further, western blotting of post-mortem brain tissue showed that the expression levels of both ALX/FPR2 and ChemR23, known SPM receptors, were upregulated in AD patients compared to non-AD controls [207]. ALX/FPR2 was significantly upregulated in glial cells whereas ChemR23 was upregulated in both glial cells and neurons. The upregulation of ChemR23 was most likely a response to lower agonist levels whereas the upregulation of ALX/FPR2 may also be due to an interaction with Aβ as discussed below.

### 7.1. Lipoxins in AD

The lipoxins represent a family of metabolites that are either directly or indirectly produced by lipoxygenases ALOX5 and ALOX12 (Figure 2). Lipoxin A_4_ (LXA_4_, (5S,6R,7E,9E,11Z,13E,15S)-5,6,15-trihydroxyicosa-7,9,11,13-tetraenoic acid) and lipoxin B_4_ (LXB_4_, (5S,6E,8Z,10E,12E,14R,15S)-5,14,15-trihydroxyicosa-6,8,10,12-tetraenoic acid) are synthesized through the production of LTA_4_ via ALOX5 which in turn is converted to both LXA_4_ and LXB_4_ by ALOX12. The 15-epi derivatives, 15-epi-LXA_4_ ((5S,6R,7E,9E,11Z,13E,15R)-5,6,15-trihydroxyicosa-7,9,11,13-tetraenoic acid) and 15-epi-LXB_4_ ((5S,6E,8Z,10E,12E,14R,15R)-5,14,15-trihydroxyicosa-6,8,10,12-tetraenoic acid) are synthesized from 15R-HETE by ALOX5 through an unstable epoxy intermediate [188]. 15R-HETE is produced from AA by aspirin inhibited (acetylated) COX-2 rather than the PGH_2_ produced by non-acetylated COX-2. Both LXA_4_ and LXB_4_ are known to be highly unstable (t_1/2_ < 30 s) in vivo and are subject to rapid dehydrogenation and reduction to a series of inactive products [208,209]. The 15-epi derivatives are two-fold more stable [210].

Only one LX receptor has been identified to date, the G-protein coupled ALX/FPR2. It is expressed in pyramidal cells neurons, as well as astrocytes and microglia [207]. This receptor binds other ligands, including peptides, and is involved in numerous inflammatory-related biological processes that are beyond the scope of this review; see the reviews by Chiang et al. [211] and Romano et al. [212]. It has been shown that both LXA_4_ and 15-epi-LXA_4_ act through ALX/FPR2 [213] with high and similar binding efficiencies (IC_50_ = 0.01–0.1 nM). LXB_4_ and presumably 15-epi-LXB_4_ act through an unknown receptor. It has been shown that the neuroprotective effect of LXB_4_ on retinal tissues cannot be blocked with specific antagonists for ALX/FPR2 and RvD2 receptors (GPR18) [214].

Each of these lipoxins has been shown to induce anti-inflammatory and pro-resolution mechanisms, including repression of leukocyte-mediated injury and pro-inflammatory cytokine production, as well as inhibition of cell proliferation and migration [213,215,216]. For example, LXB_4_, LXA_4_, 15-epi-LXA_4_, and stable analogs have been shown to block TNF-α secretion from stimulated human T cells involved in T cell-mediated inflammation [217]. In addition, in a traumatic brain injury mouse model, LXA_4_ was shown not only to inhibit TNF-α release but also other pro-inflammatory cytokines IL-1β and IL-6 while the same time it decreased BBB permeability [218]. In a complementary fashion, treatment of AD model mice with 15-epi-LXA_4_ increased levels of anti-inflammatory IL-10 and transforming growth factor-β (TGFβ) [213]. These lipoxins are also reported to be potent activators of monocytes, known to play a role in Aβ elimination, through stimulation of chemotaxis and adherence [208,219]. LXA_4_ and 15-epi-LXA_4_ have been shown to be equipotent at reversing the pro-inflammatory LTB_4_-initiated human PMN migration [210,220]. Lipoxins are also involved directly in neuroprotection. Both LXA_4_ and LXB_4_ have been reported to promote neuroprotection in retina with LXB_4_ consistently being more potent [214].

In addition to the general anti-inflammatory pro-resolving actions of lipoxins, more specific actions involving aggravation of AD progression have been reported. Treatment of AD model Tg2576 mice with 15-epi-LXA_4_ decreased the production of pro-inflammatory mediators (e.g., TNF-α, IL-1β) and at the same time increased the levels of anti-inflammatory proteins (e.g., TGFβ, IL-10) [221]. The resulting altered environment, apparently dependent on the activation of astrocytes, activated microglia into an alternative phagocytic phenotype that enhanced the clearance of Aβ plaques. In another mouse model study, LXA_4_ levels were found to decrease with age in both non-transgenic and AD-model mice and more markedly so in the latter [222]. However, restoring LXA_4_-like levels by administration of 15-epi-LXA_4_ enhanced the cognitive performance of the AD-model mice, reduced Aβ levels, and decreased levels of phosphorylated tau. The role of LXA_4_ in slowing AD progression was also confirmed in a study of CSF from AD patients and post-mortem hippocampal tissue from other AD patients where LXA_4_ levels were found to be lower than in non-AD subjects [207]. Further, LXA_4_ levels were found to correlate to mini-mental state examination (MMSE) scores, suggesting that pro-resolution eicosanoids may inhibit AD-related cognitive decline. In contrast, the ALX/FPR2 receptor levels were found to be abnormally high in these patients. This can be rationalized by the fact that there are also pro-inflammatory ligands for this receptor, including Aβ_42_ [223], and that inflammation has been shown to enhance its expression [207,224].

### 7.2. Resolvins, Protectins, and Maresins in AD

Resolvins, protectins, and maresins represent oxidized products of ω3 PUFAs that are actively involved in the resolution of inflammation. A complete description of all of these SPMs is beyond the scope of this review. Instead, the focus will be on a few classes, namely the resolvin D1 group, the protectin D1 group, resolvin E1, maresin 1, and their known and potential involvement in AD.

The resolvin D1 group is formed by a pathway similar to lipoxin pathway, where the product is formed from hydroperoxidation of docosahexaenoic acid (DHA, 4Z,7Z,10Z,13Z,16Z,19Z)-docosa-4,7,10,13,16,19-hexaenoic acid) at C-17 by either aspirin acetylated-COX-2 or ALOX15 leading to 17R-hyrdoperoxy-DHA and 17S-hydroperoxy-DHA respectively followed by reduction and then hydroperoxidation by ALOX5, enzymatic epoxidation, and hydrolysis leading to formation of the tri-hydroxy resolvins that differ only in their stereochemistry at C-17. The 17R isomer formed through the action of acetylated COX-2 is referred to as AT-RvD1 ((4Z,7S,8R,9E,11E,13Z,15E,17R,19Z)-7,8,17-trihydroxydocosa-4,9,11,13,15,19-hexaenoic acid) and the ALOX15 derived 17S as RvD1 ((4Z,7S,8R,9E,11E,13Z,15E,17S,19Z)-7,8,17-trihydroxydocosa-4,9,11,13,15,19-hexaenoic acid). Both lipoxins and resolvin D1s are enzymatically degraded by eicosanoid oxidases to biologically inactive substances. RvD1, however, has a reported half-life about four times that of LXA_4_ (<10 min) and AT-RvD1 was shown to be many times more stable yet [225]. As observed for LXA_4_ and 15-epi-LXA_4,_ both resolvins have been shown to be equally effective at reducing polymorphonuclear leukocyte (PMN) infiltration in vivo and as effective as the anti-inflammatory indomethacin [226]. It has been determined that RvD1 activates the LXA_4_ receptor ALX/FPR2 as well as GPR32 [227]. Receptors specific for AT-RvD1 have yet to be reported.

Analysis of CSF obtained from AD-patients revealed that the levels of RvD1 correlate well with MMSE scores [207]. Incubation of human microglial cells (CHME3) with RvD1 significantly reduced the Aβ-induced inflammation in human microglia and the Aβ-induced increase in CD40 production [228]. This reduction is particularly important in AD, as the concentration of CD40 has been reported to be increased in AD brain and, when bound to its ligand, promotes cell death and the production of Aβ.

Resolvin E1 (RvE1, (5S,6Z,8E,10E,12R,14Z,16E,18R)-5,12,18-trihydroxyicosa-6,8,10,14,16-pentaenoic acid) is synthesized from eicosapentaenoic acid (EPA, (5Z,8Z,11Z,14Z,17Z)-eicosa- 5,8,11,14,17-pentenoic acid) in a manner like that of AT-RvD1. EPA is hydroperoxidated by either acetylated-COX-2 or cytochrome P450 to an 18R peroxy derivative which is subsequently reduced and then hydroperoxidated by ALOX5, followed by dehydration and then hydrolysis to form the final product. A minimum of four pathways have been identified for in vivo degradation of RvE1 with at least one of the products retaining at least some of the RvE1 activities [229]. An unpublished clinical study revealed an overall half-life for RvE1 of 7 h [230]. RvE1 was shown to be twice as effective as indomethacin in reducing PMN infiltration [227] making it about twice as effective as the resolving D1 group. There are two known receptors for RvE1, the G-protein coupled ChemR23 and BLT1. The ChemR23 receptor is similar in action to that of the ALX/FPR2 receptor in that it interacts with eicosanoid and peptide ligands, the former shown to result in a reduction in PMN migration [231]. This receptor is abundantly expressed in monocytes in lower amounts in neutrophils and T lymphocytes [231] as well as in pyramidal cells, neurons, astrocytes, and microglia [207]. Binding of RvE1 to the BLT1 receptor has been shown to attenuate the pro-inflammatory action of the LTB_4_ agonist rather than elicit any specific stimulatory effect itself [192].

Treatment of AD model mice (5xFAD) with either RvE1 or LXA_4_ reduced the concentrations of Aβ_40_, Aβ_42_, and inflammatory cytokines and chemokines, the latter reduced to levels in control mice [232]. When given in combination, additional improvements were observed, including reduced activation of microglia and astrocytes and a decrease in the extent of Aβ pathology.

Protectin D1/neuroprotection D1 (PD1/NPD1, (4Z,7Z,10R,11E,13E,15Z,17S,19Z)-10,17-dihydroxydocosa-4,7,11,13,15,19-hexaenoic acid) is synthesized from DHA through hydroperoxidation by ALOX15 followed by a reduction and then hydrolysis to the final product. The half-life and degradation pathway(s) have not been reported. NPD1 has been shown to be nearly 50% more effective than either RvD1 or 15-epi-LXA_4_ in reducing PMN migration. Thus far only one NPD1 receptor has been identified, the G-protein coupled 37 receptor (GPR37) that is found in macrophages, but not microglia [233].

Treatment of aging human brain cells with NPD1 has been shown to reduce Aβ peptide release [234]. Treatment of human neuronal-glial (HNG) cells with NPD1 challenged with Aβ_42_ or transfected with βAPPsw (Swedish double mutant APP695swe) reduced the Aβ_42_-triggered expression of COX-2 and the pro-inflammatory element B94 [234]. This down-regulation of COX-2 and B94 was facilitated through suppression of β-secretase-1, activation of α-secretase and up-regulation sAPPα. The net effect is a switching from the amyloidogenic to the non-amyloidogenic pathway [235]. Further, treatment of human fetal brain tissue HN cells with NPD1 repressed Aβ_42_ activation of pro-inflammatory genes and at the same time upregulated antiapoptotic genes encoding Bcl-2, Bcl-xl, and Bfl-1(A1), thus promoting cytoprotection [236]. Interestingly, it was also shown that sAPPα, known to be upregulated by NPD1, also stimulated the biosynthesis of NPD1, thus amplifying the effect.

Maresin 1 (MaR1, (4Z,7R,8E,10E,12Z,14S,16Z,19Z)-7,14-dihydroxydocosa-4,8,10,12,16,19-hexaenoic acid) is also synthesized from DHA through hydroperoxidation by either ALOX15 or ALOX12 followed by a reduction and then hydrolysis to the final product. The half-life and degradation pathway(s) have not been reported. Reports have indicated that MaR1 may be the most potent of the SPMs in resolution of inflammation [228]. A specific receptor for MaR1 has yet to be reported.

Incubation of human microglial cells (CHME3) with MaR1 significantly reduced the Aβ-induced inflammation in human microglia and the Aβ-induced increase in CD40 production [228]. Further, of all the SPMs tested, only MaR1 increased, and significantly so, the phagocytosis of Aβ_42_. MaR1 has also been shown to downregulate pro-inflammatory markers CD11b-activated, MHC-II, CD86, and CD33, but had no effect on the anti-inflammatory markers CD163 and CD206.

## 8. Conclusions

Eicosanoids are clearly paramount to AD pathology, be it promoting progression of the disease or resolution in the early phases. It is also apparent that disruption of normal inflammation homeostasis is at the center of AD progression and appears to be the most reversible aspect of early AD. A better understanding of inflammation resolution and how to promote and control it is of major import to developing treatment regimens for AD. As shown in the preceding paragraphs, the majority of eicosanoid metabolites are related in some way to the onset, progression, or resolution of AD. A brief synopsis of their involvement is presented below.

Prostanoid biosynthesis begins with the production of free AA through the action of PLA_2_s on AA-containing phospholipids. Studies have shown that AA itself has a detrimental effect on neuronal viability and participates in a yet-to-be understood synergy with amyloid peptides [22,23,27]. Cyclooxygenases (COX-1 and COX-2) catalyze the first reaction of the prostanoid pathway to produce PGH_2_, the major precursor to other prostanoids. Numerous studies have shown that inhibition of COX-1 and COX-2 by NSAIDS can reduce the risk of AD [35,36,37,38], however, not all studies have shown such positive results [39,40] and the effect may depend on the parameters employed in each study, such as the NSAIDS used, the stage of AD when treatment was begun, as well as the diversity of the population involved in the study. More focused and carefully controlled studies would shed more light on this subject. Interestingly, the NSAID aspirin acetylates COX-2 resulting in a dramatic change in its activity that can increase the production of resolvins that are known inflammation resolving mediators and have been shown to reduce the production of Aβ peptides [232], another factor not considered in the aforementioned studies.

Eicosanoids can be crudely categorized with respect to their influence on AD in the following manner: (1) pro-inflammatory and AD promoting, (2) anti-inflammatory and AD resolving, and (3) mixed effects depending on context or when conflicting data was observed. TXA_2_, isoprostanes, 5-HETE, LTB_4_, and the cystenyl leukotrienes (LTC_4_ and LTD_4_) clearly fall into fall into the first category as they have been shown to promote Aβ production [174,189]. On the other hand, studies have shown that the involvement of PGF_2α_ in AD appears to be indirect and only slightly AD promoting, in particular, in very early stages of AD [149,150,151,152]. This may very well be due to the large number of PGF_2α_ isoforms that signal through a variety of different G protein-mediated pathways and hence may promote different intracellular processes. Lastly, the eoxins are known pro-inflammatory effectors and thus may be involved in AD progression, however, their direct involvement in AD progression has yet to be demonstrated.

The second category consists of PGI_2_, the lipoxins, the resolvins, the neuroprotectins, and the maresins. PGI_2_ has been reported to promote the production of the neuroprotective sAPPα and to upregulate the anti-inflammatory receptor PPARγ, clearly promoting resolution of AD [91]. The lipoxins, LXA_4_, LXB_4_, and 15-epi-LXA_4_ have been shown to reduce the secretion of pro-inflammatory mediators such as TNFα, while LXA_4_ has also been shown to promote the release of anti-inflammatory factors [221,222,223]. The more direct AD resolving properties of LXA_4_ and 15-epi-LXA_4_ have been demonstrated by their ability to reduce Aβ and phosphorylated tau levels [222]. Resolvin D1 and maresin 1 have been shown to reduce Aβ-induced inflammation in microglia and CD40 production, a factor known to enhance production of Aβ [228]. Resolvin E1 has been reported to attenuate the pro-inflammatory effects of LTB_4_ and reduce the concentrations of Aβ peptides [232]. Lastly, protectin D1 has also been shown to reduce Aβ concentrations while at the same time increasing concentrations of the neuroprotective sAPPα [236].

The third category is the most diverse of the three and deserves much further study. PGE_2_ is generally considered to be the prime pro-inflammatory prostaglandin. Indeed, it has been demonstrated that stimulation of EP_1_ and EP_2_ receptors by PGE_2_ reduces neural survivability, enhances Aβ production, and reduces microglial phagocytosis [106,107,108,109,110]. Stimulation of the EP_3_ receptor has been reported to mildly support the progression of AD, however, its involvement is complicated by the number of different isoforms that utilize different G protein mediations [113]. In contrast, stimulation of the EP_4_ receptor has been shown to produce an anti-inflammatory response as well as induction Aβ phagocytosis [118,119,120], and thus the overall effect of PGE_2_ is dependent on the relative expression of the different receptors. The function of PGD_2_ has also been shown to be receptor dependent, where stimulation of DP_1_ favors neuronal rescue and stimulation of DP_2_ promotes neuronal loss [56,64]. Clearly, receptor expression is another area requiring further study. The effect of the J_2_ series of prostaglandins, 15-HETE and 12-HETE, on AD progression is not understood. Some reports have shown that they enhance Aβ formation, suggesting promotion of AD progression [72,75]. On the other hand, other reports have indicated that all of these are ligands for the anti-inflammatory PPARγ receptor, suggesting quite the opposite [76,77,78,79,80]. Certainly, more work needs to be done to determine what conditions favor one path over the other.
